# Functionalized Nanomaterials for Inhibiting ATP-Dependent Heat Shock Proteins in Cancer Photothermal/Photodynamic Therapy and Combination Therapy

**DOI:** 10.3390/nano14010112

**Published:** 2024-01-02

**Authors:** Thejas P. Premji, Banendu Sunder Dash, Suprava Das, Jyh-Ping Chen

**Affiliations:** 1 Department of Chemical and Materials Engineering, Chang Gung University, Kwei-San, Taoyuan 33302, Taiwan; thejaspremji@gmail.com (T.P.P.); banendusunder@gmail.com (B.S.D.); supravadas0603@gmail.com (S.D.); 2 Craniofacial Research Center, Chang Gung Memorial Hospital at Linkou, Kwei-San, Taoyuan 33305, Taiwan; 3 Department of Neurosurgery, Chang Gung Memorial Hospital at Linkou, Kwei-San, Taoyuan 33305, Taiwan; 4 Research Center for Food and Cosmetic Safety, College of Human Ecology, Chang Gung University of Science and Technology, Taoyuan 33305, Taiwan; 5 Department of Materials Engineering, Ming Chi University of Technology, Tai-Shan, New Taipei City 24301, Taiwan

**Keywords:** photothermal therapy, photodynamic therapy, nanomedicine, cancer combination therapy, heat shock protein

## Abstract

Phototherapies induced by photoactive nanomaterials have inspired and accentuated the importance of nanomedicine in cancer therapy in recent years. During these light-activated cancer therapies, a nanoagent can produce heat and cytotoxic reactive oxygen species by absorption of light energy for photothermal therapy (PTT) and photodynamic therapy (PDT). However, PTT is limited by the self-protective nature of cells, with upregulated production of heat shock proteins (HSP) under mild hyperthermia, which also influences PDT. To reduce HSP production in cancer cells and to enhance PTT/PDT, small HSP inhibitors that can competitively bind at the ATP-binding site of an HSP could be employed. Alternatively, reducing intracellular glucose concentration can also decrease ATP production from the metabolic pathways and downregulate HSP production from glucose deprivation. Other than reversing the thermal resistance of cancer cells for mild-temperature PTT, an HSP inhibitor can also be integrated into functionalized nanomaterials to alleviate tumor hypoxia and enhance the efficacy of PDT. Furthermore, the co-delivery of a small-molecule drug for direct HSP inhibition and a chemotherapeutic drug can integrate enhanced PTT/PDT with chemotherapy (CT). On the other hand, delivering a glucose-deprivation agent like glucose oxidase (GOx) can indirectly inhibit HSP and boost the efficacy of PTT/PDT while combining these therapies with cancer starvation therapy (ST). In this review, we intend to discuss different nanomaterial-based approaches that can inhibit HSP production via ATP regulation and their uses in PTT/PDT and cancer combination therapy such as CT and ST.

## 1. Introduction

Cancer, characterized by the aberrant proliferation of healthy cells that lose their growth control mechanisms, has emerged as a very concerning and pervasive ailment in the 21st century, posing a lifelong danger to affected individuals [1]. The manifestation of symptoms in cancer often occurs at the advanced stage of the disease or when there is a notable rise in tumor size [2]. The therapeutic modality for cancer exhibits variability, which is contingent upon the specific anatomical site and dimensions of the neoplastic growth [2]. The commonly used cancer therapeutic approaches include radiation, surgery, and chemotherapy [3]. Each of these treatment modalities is associated with distinct adverse effects such as pain, nausea, vomiting, and/or distress. Consequently, in recent decades, many treatment options have been developed, including the substitution of traditional therapeutic methods with a nanotherapeutic approach with the advance of nanomedicine [3]. Moreover, there has been a growing focus on the investigation of non-invasive and selective treatments within the realm of medicine. Among these, light-activated cancer therapy has emerged as one of the most prominent therapeutic procedures, which encompasses two specific methods: photothermal therapy (PTT) and photodynamic therapy (PDT) [4]. Historically, the light-based treatment has been advocated for over a millennium, as shown by historical records indicating its use by ancient Egyptians, Indians, and Chinese to treat many ailments, including skin cancer [5].

The conventional mechanism for PTT involves exposing a photothermal agent (PTA) to a light source of suitable wavelength after its intracellular uptake by cancer cells, which can transform the light energy into heat to elicit cell apoptosis/necrosis [6,7]. The potency of this treatment is dependent on whether the light intensity is sufficient to reach the tumor and whether the generated heat can ablate tumors while leaving the surrounding tissues unharmed. Although a high-power laser light can penetrate deep into cancer tissues for tumor ablation with the help of a PTA showing reasonable photothermal conversion efficiency, the PTT unavoidably also leads to a negative impact on surrounding healthy tissues [8,9]. On account of this, mild-temperature PTT with temperatures up to 45 °C has been suggested for overcoming this dilemma. Nonetheless, mild-temperature PTT can still raise a different dilemma emerging from the induction of uplifted tumor thermal tolerance [10]. This trait of thermal resistance shown by cancer cells is associated with their self-defense mechanism, which is attributed to the heat stress-triggered generation of heat shock proteins (HSP), mainly the heat shock protein 90 (HSP90) and the heat shock protein 70 (HSP70) [11,12]. When the body temperature is raised ~5 °C above normal, the overproduction of a family of HSP molecular chaperones can assist the cells to withstand the heat stress they experience [13].

Furthermore, many reports indicate tumor cells also upregulate HSP70 production during PDT [14,15,16]. In addition, cell surface expression and release of HSP can be induced by PDT, as they are related to inflammatory and immune responses. This effect can influence the response of tumors to PDT and limit the therapeutic outcomes of PDT [17]. Therefore, inhibiting the production of HSP by cancer cells would benefit PDT in addition to PTT. The PDT is highly dependent on the participation of oxygen to yield cytotoxic reactive oxygen species (ROS). In PDT, a photosensitizer (PS) absorbs the light radiation and undergoes a photochemical reaction to generate singlet oxygen from molecular oxygen. However, the distinct hypoxic tumor environment can limit the efficacy of PDT. Thus, to improve the efficacy of PDT in combating tumors, the combination of PDT with PTT would be a reasonable choice, which depends on the downregulation of HSP production [18,19]. To accomplish this, blocking the ATP-binding sites of HSP70 and HSP90 emerges as a reasonable approach. Alternatively, deprivation of glucose supply to cancer cells can also inhibit the production of HSP. This arises as the expression level of HSP is closely related to the ATP level in the cells, which is generated during cell metabolism by using glucose as an energy source.

This paper gives an overview of different nanomaterial-based approaches for enhancing the treatment efficacy of PTT/PDT through ATP-dependent downregulation of HSP. Furthermore, the facile combination of PTT and/or PDT with other forms of cancer therapy for combination cancer therapy is also discussed. We first summarize the mechanisms and limitations of PTT and PDT, the function of HSP, and the roles HSP plays in PTT/PDT. We then provide an up-to-date overview of the nanomaterial-based approach to deliver small molecular inhibitors for regulating the activity of HSP and glucose oxidase for regulating the metabolic pathway, which can be used to increase the efficacy of PTT/PDT or in combination cancer therapy.

## 2. Photothermal and Photodynamic Therapy

### 2.1. Photothermal Therapy (PTT)

Heat was used in breast cancer therapy dating back to 1700 BC [20]. Subsequent research efforts saw the emergence of different methods to induce heat generation, including radiofrequency [21,22,23,24], microwaves [25,26,27,28], and ultrasonic waves [29,30,31]. In addition to these methods for heat generation, a promising cancer treatment modality involves the photothermal conversion of absorbed near-infrared (NIR) light by chromophores into heat for tumor ablation. This process, known as photothermal therapy (PTT), can selectively induce cancer cell death in targeted areas while sparing untargeted tissues [32]. This cancer therapeutic strategy has gathered significant attention and acceptance in the medical field, with its known minimal invasiveness and effectiveness [33]. It is worth noting that significant progress has been made in PTT, particularly with the emergence of the combination use of light-activated nanoparticles and laser light, which can enhance the efficacy of PTT by targeting tumors [34,35]. A targeted PTT approach is important since, although human tissues exhibit high molar extinction coefficients in the visible light wavelengths only, PTT with NIR light can still elicit adverse side effects in healthy cells if not targeted toward cancer cells [36,37]. In PTT, a photothermal agent (PTA) absorbs NIR light, which leads to the excitation of electrons within the PTA. Consequently, via a non-radiative decay process, energy is emitted in the form of heat. This heat, in turn, induces thermal ablation of surrounding tissues in proximity to the area where the PTA accumulates [38]. Therefore, by locating a PTA at a targeted site, the increase in temperature within a tumor can result in hyperthermia conditions for tumor ablation. Several factors affect the efficacy of PTT, such as the photothermal stability, the photothermal conversion efficiency of the PTA, and the power density of the light source [39].

### 2.2. Photodynamic Therapy (PDT)

The progression of photodynamic treatment starts from its inception in the early 1900s and is a subject of considerable interest for researchers. The historical development commences with the contributions of Oscar Raab, a German student who initially proposed the notion that when light of specific wavelengths is combined with the chemical compound acridine, it can possess the ability to eliminate infusoria. Subsequently, after a span of three years, Herman von Tappeiner and A. Jesionek documented the finding that similar treatment can also effectively treat skin cancer, which was subsequently designated as “photodynamic action” [5]. In 1975, Thomas Dougherty disclosed a key breakthrough in PDT with the injection of a hematoporphyrin derivative (HpD). In the following year, Kelly and Snell reported the therapeutic effectiveness of HpD in human bladder cancer [40]. As a refined variant of HpD, the photosensitizer Photofrin^®®^ has received clinical approval for PDT, although it is associated with the detrimental consequence of causing chronic photo-irritation to the skin [41]. Along this line, PDT remains a minimally invasive approach that utilizes light-based techniques for the treatment of oncogenic conditions as well as other diseases in fields like dermatology, urology, and gynecology. The three essential components of PDT are photosensitizer (PS), oxygen, and light irradiation. When these three elements are combined, a photochemical reaction is activated, which leads to the production of cytotoxic singlet oxygen (^1^O_2_). For effective PDT in cancer treatment, the PS should be accumulated at the tumor site and exposed to a light source of a suitable wavelength, with which the PS can generate ^1^O_2_ to break down tumor vasculature and induce the death of cancer cells [42]. As the optical window of tissue is between 600–800 nm, light of a higher wavelength generally penetrates deeper into tissues; however, exposing a PS to a light source over ~800 nm may result in unsatisfactory outcomes in PDT [43].

Other than the anticancer property, the ROS generated from PDT have additional effects on pathogens like bacteria, fungi, viruses, parasites, and injured tissues. These are referred to as photodynamic inactivations (PDI). The PDI can curb the usual resistance possessed by different classes of microorganisms towards antibiotics [44]. Studies have proposed their role in viral infections like human papillomavirus infections (HPV) and SARS-CoV-2. The study on COVID-19 indicates the generated singlet oxygen from PDT can attack and destroy the guanine residues and amino acids like histidine, tryptophan, and tyrosine, which are ideal properties present in PDT for combating SARS-CoV-2 [45,46]. However, the use of PDI is not that common due to certain limitations in designing specific PS that can perform a particular function selectively [47].

**Figure 1:**
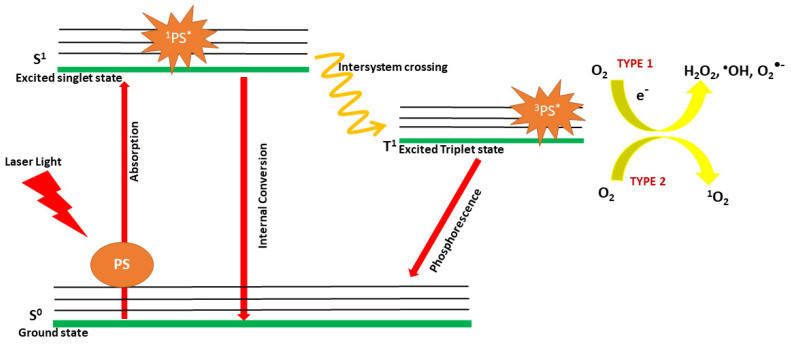
A schematic illustration of the type I and type II photochemical reaction mechanisms of photodynamic therapy (PDT). PS, photosensitizer; ^1^PS*, photosensitizer in a singlet excited state; ^3^PS*, photosensitizer in a triplet excited state.

The mechanism of PDT is a combination of photophysics and photochemistry [43]. The basic working mechanism is explained by the Jablonski diagram, a three-level energy diagram depicting light absorption, fluorescence emission, and phosphorescence emission [48]. On irradiation with light of a specific wavelength, a pair of electrons with an opposite spin in the stable ground state (S^0^) of a PS can be excited to the unstable singlet level (S^1^) by absorbing photons. In certain cases, the electrons at the S^1^ level will dissipate the excess energy via internal conversion (IC) and vibrational relaxation, thus leading to fluorescence emission. Alternatively, the PS with electrons in the S^1^ level can undergo intersystem crossing (ISC) by encountering a change in spin multiplicity to the excited triplet state (T^1^). At this T^1^ state, the PS can undertake two pathways: the radiative type decay (Type II) or the non-radiative type decay (Type I). Type II is the simplest and most important functioning mechanism in PDT, where the direct transfer of energy from the PS to the molecular oxygen existing in its basic triplet energy level (^3^O_2_) occurs, hence leading to the generation of singlet oxygen (^1^O_2_). In Type I, a transfer of hydrogen or electron occurs between the PS and innate substrates in the tissue microenvironment, leading to the formation of reactive oxygen species (ROS), followed by the production of H_2_O_2_ and superoxide anion radicals (O_2_^•−^), which are both cytotoxic (Figure 1) [42,43,49]. However, in the case of organic PS, the electronic transition to T1 is forbidden, which indeed interrupts the ISC. Recent studies focus on designing photosensitizers with advancements in raising the efficiency of the S^1^-T^1^ transition or by reducing the band gap between the energy levels [50]. One such alternative is photosensitizers based on aggregation-induced emission (AIE), which have marked proficiency in cancer therapy by generating surplus ROS through Type I non-radiative decay [51,52]. In addition to AIE luminogens, heavy metal-based PSs are also in use but restricted by their dark toxicity.

### 2.3. Factors Limiting Photothermal and Photodynamic Therapy

Several factors limit the efficacy of PTT and/or PDT. A few of these include hypoxia-induced resistance, inability to treat deep tumors, and aggregation-caused quenching in PDT [53,54,55,56]. For PTT, factors like restricted tissue permeating ability and the ablation of normal tissues when high-power laser light is used may adversely affect the efficiency of PTT [57,58,59,60]. All these factors contribute substantially to the limitation of the success of PTT/PDT, and considerable efforts were made to conquer these problems in many studies [8,61,62,63,64,65].

One major issue confronting PTT is the overexpression of HSP, which reduces the treatment efficacy due to the increased thermal resistance of cancer cells [66]. In normal tissue, heat stress to the skin can be induced by exposure to solar rays, which increases skin temperature and induces cells to produce HSP that can protect them from damage. Transient receptor proteins transient receptor potential vanilloid 1 (TRPV1) and transient receptor potential ankyrin 1 (TRPA1) are involved in the two prominent signaling pathways in skin cells to produce HSP upon NIR exposure to protect the skin tissue [67]. However, for light-induced PTT and/or PDT, we should prevent thermal-induced resistance by cancer cells by blocking the upregulation of HSP to increase the treatment efficacy.

The main targets of PDT include tumor cells, the microvasculature of the tumor bed, and the inflammatory and immune host cells. Although photodamage-linked cancer cell death can reduce cancer cell numbers, certain aspects have made it insufficient to completely eradicate tumors [41]. In the case of using PDT alone, the PS is highly dependent on oxygen for the production of cytotoxic singlet oxygen. However, due to the high proliferation ability of cancer cells, they require more oxygen during growth, thus causing a hypoxic condition in the tumor microenvironment (TME), which restricts the ability of a PS to produce ^1^O_2_ [68,69]. By combining with PTT, this bottleneck faced by PDT can be lifted, as heat generation during PTT can increase the rate of blood flow through the blood vessels, thus raising the oxygen level in the TME. In addition, hyperthermia-induced cellular activity from PTT can elevate the cellular uptake of PS and raise its accumulation rate within the tumor [70].

## 3. Heat Shock Proteins (HSP)

When the chromosomes of Drosophila salivary gland cells were subjected to elevated temperatures, puffed genes were found [71]. Later, it was found that when cells undergo heat stress, they produce certain proteins, and the general characteristics of these proteins, from bacteria to humans, were confirmed to be the same [72]. In addition to the heat stress experienced by cells, other factors like the presence of heavy metals and cytotoxic organic molecules, oxidative stress, and other environmental stresses (inflammation, tissue damage, ischemia, and reperfusion) can also induce stress and lead to the formation of HSP [73]. The HSP are highly conservative proteins present in prokaryotes and eukaryotes. Different HSPs can be found in different cell compartments like the endoplasmic reticulum, mitochondria, and cytosol with distinct functionality [74]. The HSP also help to maintain cell homeostasis and repress protein denaturation [75]. Many HSPs function as molecular chaperones and significantly contribute towards a mechanism for cells to defend themselves against damage caused by misfolded, impaired, aggregated, or insoluble proteins, which can lead to toxic inclusion bodies and aggresomes. This property associated with the HSP is favorable from a cell-protective point of view but has a negative impact in the case of cancer treatments [76]. In eukaryotes, HSP production in the transcriptional stage is maintained by heat shock elements and heat shock factors. The heat shock factor is a transcriptional regulator with the ability to bind to a particular -nGAAn- pentameric repeat DNA sequence present in the heat shock element and is responsible for inducing heat stress and finally leading to HSP gene expression [77].

### 3.1. Types of Heat Shock Proteins

According to the molecular weight, the HSP is classified into different types: HSP60 with a 60KDa molecular weight, HSP70, HSP90, HSP100, and small HSP (sHSP). The major chaperone functions in the cytoplasm are performed by HSP70 and HSP90 [78]. Among the family of HSP, members other than sHSP are ATP-dependent molecular chaperones. HSP70 is involved in cell growth and proliferation, and erythroid differentiation. It is involved in the folding and refolding of new and misfolded proteins, precursor protein translocation from the cytosol to the mitochondria, and breakdown of denatured proteins, as well as acting as a carrier of proteins crossing the membranes [78,79]. This 70 kDa protein chaperone predominantly focuses on leucine-bound small hydrophobic peptide sequences in the initial stage of protein folding. In contrast, the HSP90 chaperone sees its part of action during the late stage of protein folding, where, due to their large binding region, only multiple low-affinity contacts were created [80].

**Figure 2:**
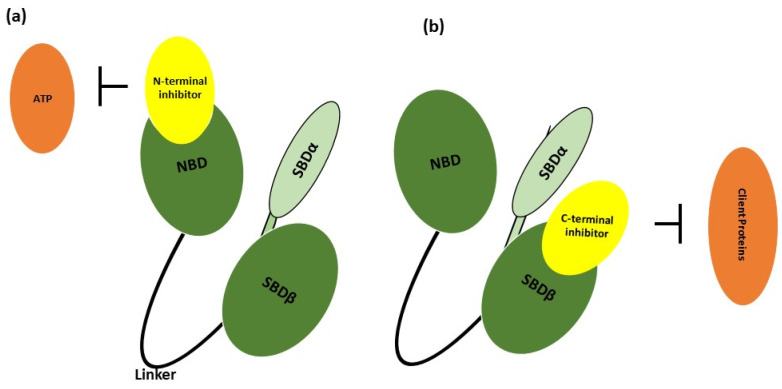
A schematic diagram of HSP70 and the inhibition of HSP70 via the N-terminal and C-terminal inhibitors. The HSP70 has an N-terminal nucleotide-binding domain (NBD) responsible for ATPase activity and a C-terminal substrate-binding domain (SBD) required for peptide binding. A linker connects two domains. The N-terminal NBD provides ATP/ADP pockets for ATP binding. The SBD is further divided into two subdomains, SBDα and SBDβ, which function as the lid and core, respectively, during substrate binding. (**a**) The N-terminal inhibitor binds to NBD by competing with ATP, thus hindering its interaction with the NBD of HSP70. (**b**) The C-terminal inhibitor binds to SBDβ by restricting the interaction of HSP70 with its client proteins.

The framework of HSP70 comprises a C-terminal substrate-binding domain (SBD) with two functional parts: SBDα (acts as a lid during substrate binding) and SBDβ (acts as substrate binding core) (Figure 2). It also has a conserved Glu-Glu-Val-Asp (EEVD) motif at the end of the C-terminal to bind with the TPR repeat units and an N-terminal binding domain (NBD) involved in ATP hydrolysis upon ATP binding. Both are connected with the assistance of a linker, which carries out the responsibility of altering the conformation during ATP binding to the NBD [81,82]. Signal transduction from NBD to SBD occurs once after the hydrolysis of the nucleotide at the NBD, and this signal that reaches SBD controls the SBDα movement (lid) and the SBDβ provides hydrophobic surroundings for the substrate binding [74]. HSP70 exists either in ATP-bound (open) form or in ADP-bound (closed) form, but HSP70 prefers to be in its open form due to its high affinity for ATP and hence maintains a weak binding with the substrate [83].

**Figure 3:**
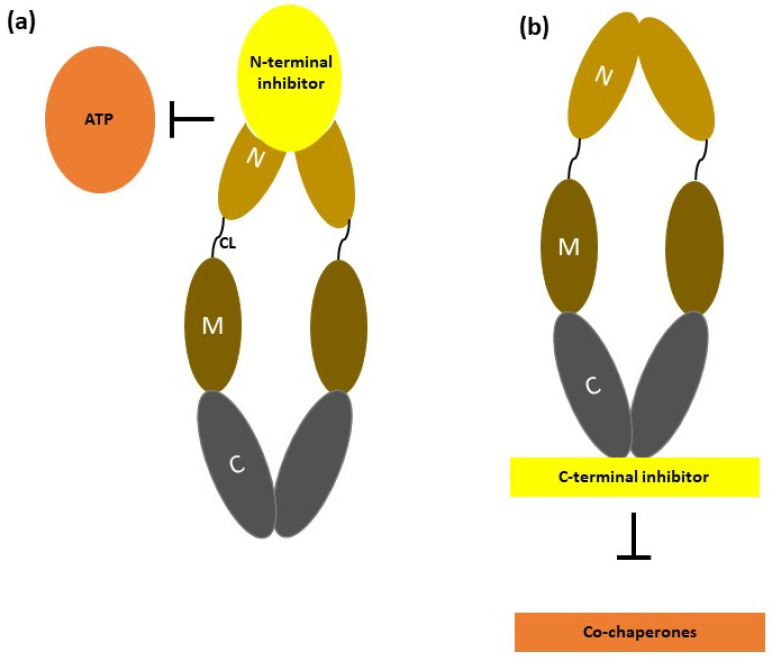
A schematic diagram of HSP90 and the inhibition of HSP90 via the N-terminal and C-terminal inhibitors. The N, M, and C stand for the N-terminal domain (NTD), middle domain (MD), and C-terminal domain (CTD) in HSP90, respectively. The NTD and MD are connected by the charged linker (CL). (**a**) The N-terminal inhibitor binds to the NTD of HSP90 and halts its interaction with ATP. (**b**) The C-terminal inhibitor binds to the CTD of HSP90 and blocks its interaction with the co-chaperones.

The structural framework of HSP90 consists of 3 main domains: the N-terminal domain (NTD) serves its function in ATP binding, the middle domain (MD) jointly with NTD performs ATP hydrolysis, and the C-terminal domain (CTD) performs dimerization, which is also bridged to the Met-Glu-Glu-Val-Asp (MEEVD) motif that enables interaction with the subset of TPR containing cochaperones [84,85] (Figure 3). Yet another structural element present uniquely in eukaryotic HSP90 is a linker connecting NTD with MD named charged linker (CL) [86]. Even though the role is unclear, this linker is comprised of multiple amino acids that provide conformational flexibility in the NTD–MD interface and, as a result, ameliorates the interaction of NTD with the client proteins [87]. In addition to this, NTD and CTD show specificity while binding with the ligands; NTD mainly binds to adenosine nucleotides, while CTD binds either to purine or pyrimidine nucleotides [88]. Research has affirmed the importance of dimerization of HSP90 in ATP hydrolysis [89]. HSP90 prefers to be in an open apo conformation, which is followed by dimerization and NTD movement upon binding of ATP to the NTD, which further transforms HSP90 into a closed conformation [90]. In the case of an unbound HSP90 in its open conformation, the unfolded client proteins that approach HSP90 are loaded at the MD; this is followed by HSP90 dimerization on ATP binding. Further, ATP hydrolysis changes their conformation to a closed state, and upon subsequent release of the folded client protein and ADP, HSP90 regains its open form [85]. The interaction of these client proteins with the newly synthesized peptides for their folding and activation is initiated via the HSP70 interaction and followed by the transfer of these peptides to HSP90 by the HSP70–HSP90 organizing protein (Hop). In the latter stage, HSP70 and Hop dissociate from HSP90, leading to a series of functions like NTD dimerization, formation of closed conformation, and other cochaperone binding. Thus, a “mature complex” is formed comprising HSP90 and p23, an HSP90 cochaperone that assists interaction with client proteins [91]. Employing an HSP90 inhibitor capable of occupying the nucleotide binding sites can obstruct the entire cyclic process [88,92].

### 3.2. HSP70 and HSP90 Inhibition

It has been reported that HSP90 inhibition can occur by attacking its N-terminal ATP binding site or C-terminal cochaperone binding site. Most of the clinical study is focused on designing inhibitors that can mimic ATP and compete with ATP for binding at the ATP-binding site at the NTD. These inhibitors can counter the overexpression of HSP90 from heat stress induction and turn on the cellular self-defense mechanism [93]. As ATP binding and hydrolysis are major activities of HSP90, any interference could also cause hindrance to the HSP90 chaperoning ability [91,94]. Therefore, as an HSP90 inhibitor competes for the N-terminal binding site by blocking the ATP interaction, HSP90 loses its capability in protein folding and processing of client proteins. This act of inhibiting ATP binding with HSP90 NTD can induce the prosurvival heat shock response and thereby inhibit HSP70 expression [95]. An alternative to N-terminal HSP90 inhibition can also be employed by choosing modulators for the C-terminal of HSP90, as they generally do not induce HSR [95,96,97]. Even though HSP90 inhibition can successfully promote the misfolding of proteins and concomitantly remove these damaged proteins supported by proteosomes, these degraded proteins may trigger HSP70 overexpression, which limits the effectiveness of this inhibition mechanism [98]. Similarly, inhibition of HSP90 can also trigger heat shock transcription factor 1 (HSF1), which, after being self-activated, can dissociate from HSP90 and undergo trimerization after translocating into the nucleus. This HSF1 trimer identifies and binds with the heat shock element and gene transcription for themselves or other multicellular chaperones like HSP70 can occur, which is the mechanism responsible for cytoprotection [93].

HSP70 expression is immensely high on malignant cell surfaces. Clinical trial results indicate the presence of two kinds of HSP70 produced by necrotic cells and tumor cells as damage-associated molecular patterns (DAMPs) and exosomal HSP70, respectively, in the bloodstreams of cancer patients. In the initial stage of tumor growth, HSP70 is produced as a tumor suppressor, but overexpression of HSP70 in the later stage helps cell proliferation. The necrotic cells that produce DAMPs can increase the immunogenic response by functioning as an anti-tumor agent [99]. The reason for considering HSP90 as the target moiety in cancer therapeutics is due to certain key factors possessed by cancer cells towards this 90 kDa protein. The factors that force cancer cells to depend upon HSP90 include the maturation and stabilization of cancer client proteins during extreme situations like hypoxia, variation in pH, and a low source of energy in the TME. This underlines the striking existence of HSP90 as a complex multi-chaperone with a strong affinity towards ATP and drugs [100]. However, as discussed before, HSP90 inhibition alone cannot meet the expected efficacy, and additional attention toward HSP70 inhibition can increase the efficiency of inhibition.

HSP70 is a versatile chaperone that can perform multiple functions like protein translocation, cellular resistance, apoptosis inhibition, and maintenance of the functions of anti-apoptotic proteins [93,101,102]. It can modulate apoptosis at a variety of levels, ranging from death receptor signaling to those in charge of the cell death program. The activation of caspase-mediated cytoprotection via apoptosome is hindered by the intervention of HSP70 with the apoptosis signaling pathway by binding with apoptotic protease activating factor-1 (APAF-1). Thus, inducing overexpression of HSP70 becomes the key factor for amplifying the effect of HSP90 inhibitors and decreases the apoptosis rate [103]. Therefore, the necessity to inhibit HSP70 is rendered through the inhibitors similar to those of HSP90. HSP70 inhibition can be intensified either by introducing ATP competitors disrupting the ATP binding and hydrolysis at NBD or employing SBD inhibitors obstructing the cochaperone and client proteins at SBD [104,105].

## 4. Downregulation of HSP90/HSP70 through a Nanomaterial-Based Approach in Cancer Therapy

There are different ways to downregulate HSP90 and HSP70 for cancer therapy. One of them is the use of small HSP inhibitors that can deprive ATP binding by competing with the ATP-binding sites in the N or C-terminal domains. The other is to deprive the energy source for the cells by inducing cancer cell starvation. Here, we have provided overviews of previous efforts to suppress HSP production through nanomaterial-based approaches. We categorize them into indirect ATP suppression via nanomaterial-based delivery of small-molecule HSP inhibitors and direct ATP suppression via nanomaterial-based delivery of glucose-consuming enzymes.

### 4.1. HSP Inhibition via Indirect ATP Suppression with Small-Molecule HSP Inhibitors Delivered by Nanomaterials

**Figure 4:**
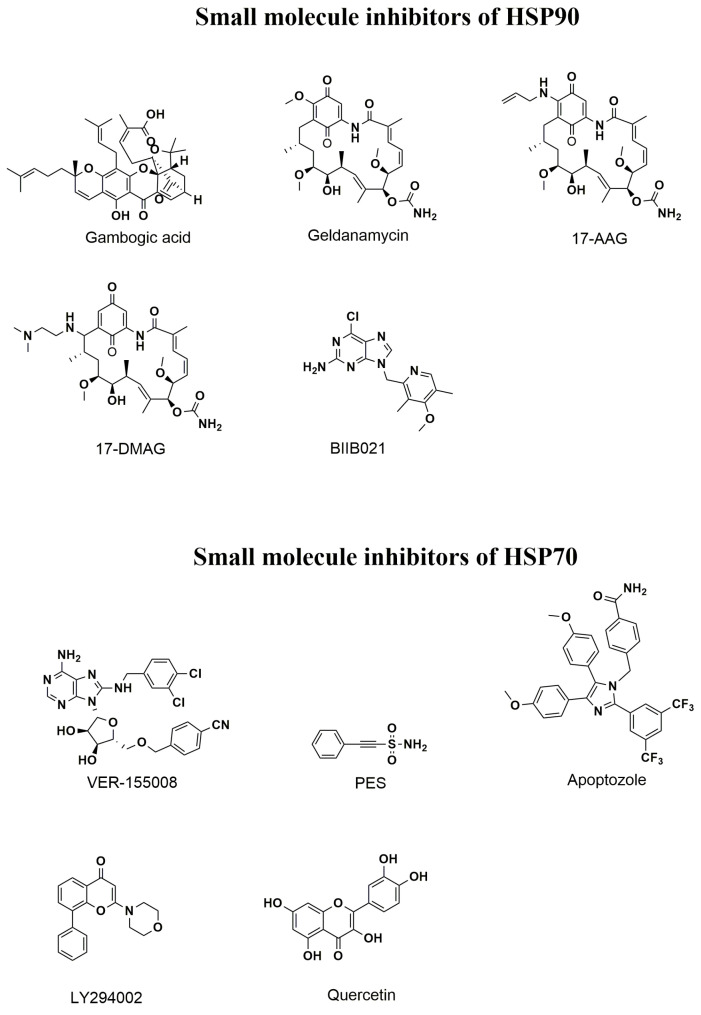
The chemical structure of small-molecule inhibitors of HSP90 and HSP70.

Nanomaterials can be used as nanocarriers for drug delivery to a specific site for cancer treatment. For HSP inhibition, delivery of small-molecule HSP inhibitors to the tumor site could be accomplished together with a PTA and/or a PS for PTT and/or PDT. However, the size of the nanocarriers must be smaller than 200 nm for them to move within microcapillaries for drug delivery. They should also have good biocompatibility, a good pharmacokinetic response, and preferably be endowed with targeting ability towards cancer cells [106]. As discussed in previous sections, the importance of HSP90 in tumor cell proliferation, differentiation, and apoptosis emphasizes choosing HSP90 as a target [107]. Towards this end, many small molecules, such as gambogic acid, geldanamycin, 17-AAG, 17-DMAG, and BIIB021, which can block the activity of HSP90 were used as natural or synthetic inhibitors to bind with the N- or C-terminal binding sites of HSP90 [108]. For HSP70, Ver-155008, PES, apoptozole, LY294002, and quercetin could be used. The chemical structure of small-molecule inhibitors of HSP70 and HSP90 are shown in Figure 4.

#### 4.1.1. HSP90 Inhibitor: Gambogic Acid (GA)

Gambogic acid (GA), a member of the xanthone family, can be extracted from the *Garcinia hanburyi* tree and works as an initiator of apoptosis in different cancer cells [109]. It is involved in upregulating p53 expression, thus leading to the inhibition of B-cell lymphoma 2 (Bcl2) expression (an anti-apoptotic protein) and MDM2 suppression in lung cancer and breast cancer cells, as well as working as a N-terminal inhibitor of HSP90 [109]. Although GA shows antitumor activity, one study cautions against the use of GA as a chemo drug as it exhibits an adverse impact by inducing a heat shock response in the presence of free thiol groups and leads to the release of heat shock factors 1 and 2 [110]. However, it is worth noting that GA can elevate ROS and deplete glutathione (GSH) to disrupt intracellular redox homeostasis, which can be used to improve PDT efficacy [111].

Sun et al. used GA in black phosphorus quantum dots (BPQDs) in combination with poly(L-lactide)-poly(ethylene glycol)-poly(L-lactide)-triblock copolymer (PLLA-PEG-PLLA) to prepare BPQDs/GA/PLLA-PEG-PLLA (BGP) for PTT [112]. The PLLA-PEG-PLLA acts as a nanocarrier, while photothermal activity is exhibited by BPQDs. GA functions as a chemotherapeutic drug as well as downregulating HSP90 production. The photothermal effect was shown to be dependent on the laser irradiation time and BGP can induce temperature elevations of 50–55 °C. An in vitro study with Western blotting concluded the importance of GA in reducing HSP90 expression to 41% and 63% in T47D and MCF-7 cancer cells, respectively, and the expression level could be further downregulated using BGP for GA delivery. With GA being a prominent inhibitor of HSP90, GA delivered by BGP was shown to take part in the signaling pathways of MAPK cascades, and an increase in GA concentration can lead to an increased inhibition level of p-ERK1/2 expression.

Sun. et al. prepared polymer-GA nanoparticles (PGNPs) by encapsulating the polymer poly((2,5-diyl-2,3,5,6-tetrahydro-3,6-dioxo-pyrrolo(3,4-c)pyrrole-1,4-diyl)-alt- (2,2′:5′,2”-ter-thiophene-5′,5”-diyl)) (PDPP3T) for photothermal response and GA for HSP90 inhibition in Pluronic F-127 [113]. Using an 808 nm laser to induce mild hyperthermia, the PGNPs could enhance the photothermal activity by dramatically blocking HSP90 overexpression and increasing the apoptosis rate of cells to promote the efficacy of PTT. The PGNPs could inhibit tumor growth in vivo compared with nanoparticles without GA and show minimum systemic toxicity.

A low-temperature PTT study with HSP90 inhibition was attempted by Yang. et al. by using poly(ethylene glycol) (PEG)-modified one-dimensional coordination polymers (NCPs), formed by mixing Mn^2+^, indocyanine green (ICG), and poly-L-histidine-PEG (pHis-PEG) copolymer, to load GA [114]. Mn-ICG@pHis-PEG/GA can induce tumor cell apoptosis with NIR-triggered mild PTT (~43 °C) by successfully downregulating HSP90 expression within tumors. By intravenous injection of Mn-ICG@pHis-PEG/GA in mice tumor models, the nanocarrier was retained in the tumor and efficiently activated the death of cancer cells, after which a renal clearance mechanism was found for the nanoagent to support its high biocompatibility.

In a different study, Gao et al. coupled GA with human serum albumin (HAS) and dc-IR825 to fabricate the nanocomposite HSA/dc-IR825/GA [115]. The author affirms, through bio-TEM and confocal laser scanning microscopy, the ability of the as-prepared nanoagent to mediate GA translocation in the cytosol via mitochondrial breakage from generated ROS upon NIR irradiation and to downregulate HSP90 expression. Using GA as an anticancer agent, the synergistic effect of mild PTT and HSP90 inhibition can lead to enhanced PTT and chemotherapy (CT).

For low-temperature PTT, Jin et al. synthesized a rambutan-like nanocomplex with a polydopamine-functionalized S-nitrosothiol (PDA–SNO) core and a GA-derivatized hyaluronic acid (HA–GA) shell for targeted delivery of doxorubicin (DOX) [116]. After endocytosis, the PSGHD nanocomplex can gradually release both DOX for chemotherapy and GA under different physiological conditions. Using 808 nm NIR light, the PSGHD nanocomplex can increase the local temperature to 43 °C for low-temperature PTT and convert SNO to NO, which can decrease DOX efflux during chemotherapy. These effects lead to an 8-fold increase in cancer cell apoptosis rate in vitro using GA to enhance thermal damage. The in vivo data showed that the nanocomplex can significantly prolong the survival rate of tumor-bearing mice for combined PTT/chemotherapy.

Li. et al. created a multifunctional nanocomposite with a core-shell structure for synergistic PTT, PDT, and chemotherapy of breast cancer [117]. By loading GA as an HSP90 inhibitor and gold nanostars (AuNS) as a photothermal reagent in self-assembled Zr^4+^/tetrakis (4-carboxyphenyl) porphyrin (TCPP) (ZrTCPP), the AuNS@ZrTCPP-GA was further coated with PEGylated liposome (LP) to prepare AuNS@ZrTCPPGA@LP with good biocompatibility and stability. The nanosized ZrTCPP metal-organic framework degrades in the acidic TME and releases AuNS, Zr^4+^, TCPP, and GA for combination cancer therapy via chemotherapy by GA, mild PTT mediated by AuNS, and PDT mediated by TCPP. The introduction of GA can also reduce the thermal resistance of cancer cells by inhibiting HSP90 and sensitizing PTT. The constructed nanoplatform demonstrated remarkable anti-tumor activity in vitro and in vivo.

**Figure 5:**
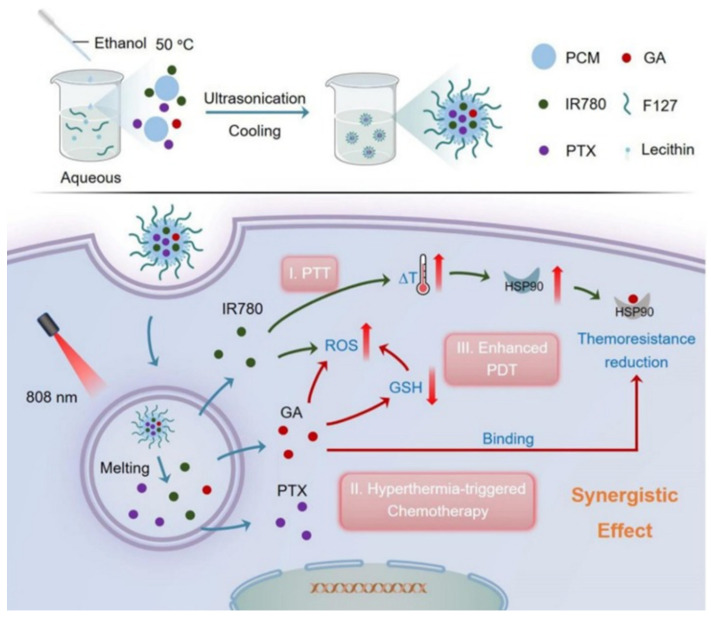
Dually enhanced phototherapy by gambogic acid and hyperthermia-activated chemotherapy for synergistic breast cancer treatment. Reprinted with permission from [118]. Copyright 2023, Elsevier B.V.

Using an ultrasonication process, Wang et al. combined the chemo drug paclitaxel (PTX), IR-780, and GA to prepare PTX-IR780-GA for PTT/PDT/chemotherapy of breast cancer (Figure 5) [118]. Using 1-hexadecanol and oleic acid, they prepared a thermoresponsive phase-change material with a melting point of 46 °C, that can be used to control the drug release triggered by hyperthermia. When exposed to NIR light, this material can be transformed from solid to liquid and release PTX and IR-780, which can produce ROS for PDT and induce PTT from mild hyperthermia. The released GA can bind to the ATP-binding pocket of HSP90 and upregulate the levels of ROS by consuming its intracellular scavenger glutathione (GSH). The ability of GA to elevate ROS and downregulate the expression of HSP90 was verified. The tumor-bearing mice administered with PTX-IR780-GA showed a 50% reduction in tumor size when compared with PTX-IR780, confirming the potential of GA to boost the efficiency of mild PTT/PDT.

As mentioned before, the therapeutic efficacy of PDT is restricted by tumor hypoxia, while PTT is limited by thermal resistance from overexpression of HSP. Therefore, Zhang et al. aimed to improve the efficacy of phototherapy (PTT + PDT) by employing self-assembled human serum albumin (HSA) nanoparticles that can combine HSP inhibition and hypoxia relief [119]. For this purpose, HSA nanoparticles that could co-deliver IR-780 and GA were prepared and surface-decorated with MnO_2_ to form IGM nanoparticles. MnO_2_ could relieve tumor hypoxia by reacting with intracellular hydrogen peroxide to produce oxygen. With NIR irradiation, the generation of ROS was significantly increased to enhance PDT, while the binding of GA to HSP90 was also promoted under irradiation to reduce heat tolerance of tumor cells to enhance PTT for mild PTT/PDT.

#### 4.1.2. HSP90 Inhibitor: Geldanamycin (GM)

Geldanamycin (GM) is a natural inhibitor of HSP90 and a member of the ansamycin derivative of benzoquinone. In the initial stage of research, it was expected that the antitumor activity of GM was exerted by inhibiting the catalytic activity of oncogenic tyrosine kinases (v-Src). Later, it was demonstrated that HSP90 inhibition by GM is the main reason for its anticancer activity through the interference of binding at the ATP-binding site of HSP90. The GM can inhibit the HSP 90 ATPase activity, which can maintain the conformation and function of oncogenic protein kinases that are responsible for the progression of the cell cycle and cell apoptosis. By fitting into the N-terminal ATP-binding pocket of HSP90, GM causes a change in the molecular conformation of HSP90 and leads to ubiquitin proteosome-dependent degradation, thus inhibiting ATP-binding and other HSP90 molecular chaperone functions [120,121].

In a recent study, Peng et al. formulated a synergistic therapeutic strategy for cancer treatment supported by mild PTT. The nanoplatform is constructed by using an NIR-triggered thermosensitive liposome (TSL) as a nanocarrier to encapsulate GM (gel) and the photothermal agent BDPII [122]. The BDPII-gel@TSL plays a dual role as a photothermal agent with 81% photothermal conversion efficiency (PCE) and produces a mild hyperthermia effect to 42 °C upon 808 nm NIR irradiation. This leads to the burst release of GM from BDPII-gel@TSL after the phase transition of TSL and leads to downregulation of HSP90 expression and inhibition of cancer cell growth.

Wen et al. used self-assembled bovine serum albumin (BSA), GM, and canine dye-7- squaraine dye (Cy7–SQ) to construct the BSA/Cy7–SQ/GM multifunctional nanoplatform. The PTA for PTT is Cy7 and the PS for PDT is SQ, which are linked covalently by sulfide bonds present in their carbon chains. This can improve thermal stability and photostability. The Cy7–SQ cleaves in the presence of GSH on reaching the TME and irradiation at 808 nm and 630 nm separately induce the production of singlet oxygen from SQ and tumor ablation from Cy7. As GM can prevent HSP90 and survivin (an anti-apoptotic protein) overexpression, GM-driven synergistic PDT/PTT could be paired with chemotherapy [123]. Although research using GM for HSP90 inhibition is still ongoing, the fact that it shows significant liver toxicity cannot be ignored. This can be addressed by preparing semi-synthetic derivatives of GM, such as 17-allylamino-17-demethoxygeldanamycin (17-AAG, tanespimycin) and 17-dimethylaminoethylamino-17-demethoxy-geldanamyci (17-DMAG, alvespimycin) by substituting the 17-methoxy group in GM with amines.

#### 4.1.3. 17-Allylamino-17-demethoxygeldanamycin (17-AAG)

The 17-AAG is a less toxic derivative of GM but shows a powerful anticancer activity similar to the parent drug, which is also the first derivative entering the clinical trial [124]. It binds to the N-terminal ATP-binding domain of HSP90 to hinder ATP-binding as well as inhibit p-ERK1/2 and p-Akt, which can prevent the formation of the HSP90 multi-chaperone complex and trigger the ubiquitin-proteasome cascade to destroy client proteins. Even though HSP90 expression occurs both in healthy and malignant cells, 17-AAG can specifically attenuate tumor growth and exhibit selectivity for cancer cells by inhibiting HSP90 expression in a multichaperone complex formed only in malignant cells [125]. Although 17-AAG has suitable pharmacological potency, it also has high hepatotoxicity and low water solubility. These drawbacks associated with 17-AAG for HSP90 inhibition may be surpassed by adopting a nanomaterial-based drug delivery approach [125].

Zhu et al. developed a multifunctional nanoplatform for mild PTT/chemotherapy with multimodal imaging ability by constructing a biodegradable disulfide-bridged hollow mesoporous organo-silica nanocapsules (HMONs) loaded with 17-AAG and ICG [126]. Gemcitabine (Gem), playing a dual role as a chemo drug and as a gatekeeper, was covalently bonded to HMONs by a pH-sensitive acetal bond that degrades in the acidic TME and releases 17-AAG and the ICG. The released ICG is used both as a PTA for PTT and as an imaging agent for NIR-fluorescence and photoacoustic (PA) imaging. To improve the pharmacokinetic and pharmacodynamic properties, NH_2_-PEG was attached to the surface of HMON to prepare ICG−17AAG@HMONs−Gem−PEG. The anti-cancer therapeutic effect was achieved not only by incorporating 17-AAG in the nanocomposite to improve the ICG-mediated PTT at low temperatures (41 °C) but also by combining with gemcitabine for chemotherapy, which can provide impressive antitumor outcomes from in vitro and in vivo experiments.

**Figure 6:**
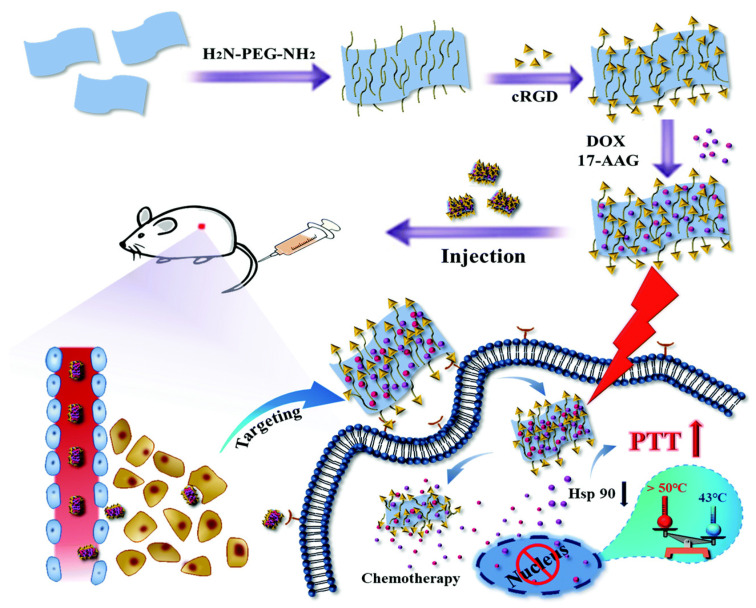
Functionalized boron nanosheets as an intelligent nanoplatform for synergistic low-temperature photothermal therapy and chemotherapy. Reproduced with permission from [127]. Copyright 2020 Royal Society of Chemistry.

Zhu et al. developed a boron (B)-based multifunctional nanoplatform by incorporating the chemotherapeutic drug doxorubicin (DOX) and the photothermal agent boron nanosheet. The B nanosheet was surface-modified with NH_2_-PEG-NH_2_ and cRGD to target the overexpressed α_v_β_3_ on the tumor cell surface for loading DOX and 17-AAG by physical adsorption [127]. The DOX-17AAG@B-PEG-cRGD was used successfully for targeted delivery of DOX and 17-AAG, where DOX-mediated chemotherapy and HSP90 inhibition-mediated enhanced low-temperature PTT from 17-AAG provide excellent anti-cancer efficacy (Figure 6). An in vivo murine cancer mode indicates the injection of DOX-17AAG@B-PEG-cRGD plus NIR laser irradiation can reduce the cell viability depending on the concentration of 17-AAG. This treatment provides a significant reduction in cell number compared to the injection of free 17-AAG or DOX-17AAG@B-PEG-cRGD without laser irradiation.

Aiming to boost penetration and accumulation of 17-AAG in tumor tissues, Yang et al. bind 17-AAG to carboxyl group-functionalized W_18_O_49_. By incorporating iRGD, a 9-amino acid cyclic peptide that binds selectively to integrin α_v_β_3_ present on tumor cells for active targeting, mild PTT with HSP90 inhibition could be achieved [128]. The in vivo results demonstrate the effectiveness of 17-AAG and establish iRGD-W_18_O_49_-17-AAG as a multifunctional nanoplatform for dual-imaging (CT and NIR imaging) nanotheranostics. The in vivo results show the targeting ability of iRGD-W_18_O_49_-17-AAG and a mild PTT effect with a temperature rise of only 10 °C, presumably due to the inhibition of the overexpressed HSP90. Dong et al. constructed a nanodrug based on (2,2-azobis[2-(2-imidazolin-2-yl) propane]dihydrochloride) (AIPH), an azo initiator capable of generating cytotoxic oxygen-independent ROS. The AIPH and 17-AAG are co-loaded with mesoporous polydopamine (MPDA), which acts as a PTA by exhibiting 25.6% PCE. The M-17AAG-AIPH could be used in mild PTT by maintaining photothermal stability without photobleaching even after three on/off laser cycles at 808 nm. The efficiency of 17-AAG in HSP90 suppression was demonstrated in both in vitro and in vivo studies [129].

In another study, Zhang et al. developed a nanodrug from gold nanorods (GNRs)-mediated mild PTT [130]. The nanoplatform was stabilized by PEGylation, followed by lactobionic acid (LA) modification to specifically target hepatocellular carcinoma cells, and finally encapsulated with 17-AAG to prepare T-GNR_AAG_ for overcoming HepG2 hepatoma cells HSP90 overexpression. Here, LA functions as a selective targeting moiety for HepG2 due to its ability to interact with asialoglycoprotein receptors expressed on the cell surface, and 17-AAG can prevent thermal resistance by inducing cell apoptosis and boosting the pharmacokinetic property of the nanodrug. The zeolitic imidazolate framework (ZIF) is one of the metal-organic frameworks for cancer treatment with its degradability in an acidic environment and the large specific surface area. Sun. et al. used ZIF to carry 17-AAG, indocyanine green (ICG), and 2,2′-azobis[2-(2-imidazolinI-2-yl) propane] dihydrochloride (AIBI) to prepare a hyaluronic acid (HA)-mediated targeted delivery system A/I@aZIF@AAG@HA (AIZAH) [131]. It can target the overexpressed CD44 receptors on the surface of prostate cancer cells. After NIR laser irradiation, ICG can produce ROS for PDT as well as heat by acting as a PTA, which leads to the decomposition of AIBI and releases oxygen-independent free radicals to augment the hypoxia-limited ROS generation from ICG. The released 17-AAG can downregulate the expression of HSP90 to achieve mild hyperthermia, which further leads to the inhibition of its client proteins (anti-apoptotic proteins and androgen receptors) and induces prostate cancer cell apoptosis to improve the efficacy of PDT. In vitro cell culture studies indicate that by combining enhanced ROS generation and 17-AAG, the nanomaterial can enhance PDT and exhibit a good anticancer effect on prostate cancer cells. To solve incomplete tumor cell elimination and uncontrollable drug release, Liu et al. developed NIR-responsive nanoparticles for 17-AAG delivery in combined PTT/PDT [132]. The nanoparticle contains an inner hydrophobic core prepared from two perylene moieties (2PMI), which are linked to diamino anthraquinone (AQ) (2PMI-AQ) as chromophores. A layer of hydrophilic thermosensitive polymer was coated to the core by spontaneous self-assembly, which allows the loading and photothermal release of 17-AAG. The 17AAG@P(2PMI-AQ) works upon a dual-sensitized mechanism upon NIR irradiation to release 17-AAG, with induced oxidative stress and diminished thermal resistance by inactivating HSP90 to enhance PTT/PDT, and successfully achieves mild tumor phototherapy in vivo.

A biodegradable nano-sized theranostic agent was developed by encapsulating 17-AAG in hollow mesoporous organosilica nanoparticles (HMONs), which are biodegradable by GSH in the TME [133]. Bovine serum albumin-iridium oxide nanoparticles (BSA-IrO_2_) surrounding the pores of the HMONs can act as a gate, which produces catalase-like activity upon reacting with H_2_O_2_ to produce O_2_ for alleviating hypoxia as well as for the pH-responsive release of 17-AAG. After modification with polyethylene glycol (PEG), the 17AAG@HMONs-BSA-IrO_2_-PEG (AHBIP) nanoparticle can be used for photoacoustic (PA)/computed tomography (CT) multimodal imaging. It also offers impressive therapeutic outcomes in vitro and in vivo with synergistic low-temperature PTT/PDT by inducing cell apoptosis at low temperatures (41 °C) and alleviating hypoxia.

#### 4.1.4. HSP90 Inhibitor: 17-Dimethylaminoethylamino-17-demethoxy-geldanamycin (17-DMAG)

DMAG is a semisynthetic derivative of GM. It shows higher water solubility, a longer plasma half-life, and less hepatotoxicity [134,135,136]. To combine HSP90 inhibition-enhanced mild-temperature PTT and chemotherapy, a biomimetic nanoplatform for 17-DMAG delivery was developed based on mesoporous platinum nanoparticles (MPNPs) [137]. The MPNPs act as a PTA and undergo biodegradation to release Pt^2+^ after reaching the acidic TME to exert a chemotherapeutic effect. The 17-DMAG is loaded onto the MPNPs to enhance PTT. The platelet membranes (PM) are coated on the surface of MPNPs for targeting cancer cells and to provide biocompatibility to synthesize 17-DMAG/MPNPs@PM. In vitro analysis confirms the cytotoxicity elicited by 17-DMAG/MPNPs@PM upon laser irradiation. In addition, the enhancement of PTT due to HSP90 suppression was confirmed through Western blotting to determine the production of HSP90 from released 17-DMAG. In vivo, experiments through tail vein injection of 17-DMAG/MPNPs@PM and 808-nm laser irradiation indicate this nanoparticle can eradicate subcutaneously implanted tumor cells from mild hyperthermia at ~41 °C.

A nanoplatform developed by Lian et al. used hollow mesoporous Prussian Blue nanoparticles (HMPB) to encapsulate 17-DMAG [138]. It was then surface-wrapped with star-PEG-PCL (sPP), a thermosensitive phase transition material, and coated with HA on its outer surface to enhance its hydrophilic nature and endow it with targeting ability. Upon laser exposure and under pH-responsive conditions, sPP stimulates the release of 17-DMAG and the ferrous/ferric ions present on the PB surface from 17-DMAG-HMPB@sPP@HA. The PTT is thus boosted as a result of inhibition of the HSP90 signaling pathways, where expression of the downstream proteins cyclin A2, protein kinase Akt, phosphorylated-Akt, and hypoxia-inducible factor HIF-1 could be verified from Western blot analysis. Additionally, this also aids in elevating the ferroptosis by increasing the ROS level for PDT.

**Figure 7:**
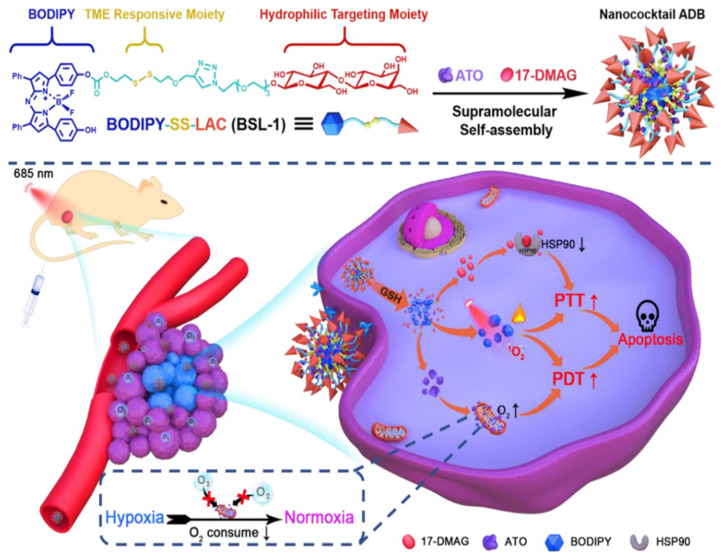
A nanoagent based on supramolecular glyco-assembly for eradicating tumors in vivo. Reproduced with permission from [139]. Copyright 2022 American Chemical Society.

Using boron-dipyrromethene (BODIPY) as a PS, Feng et al. prepare glycosylate BODIPY by linking the hydrophobic BODIPY and the hydrophilic lactose moiety via GSH-responsive disulfide bonds. The synthesized BODIPY-SS-LAC (BSL-1) can target hepatoma cells. A supramolecular self-assembly approach was used to prepare a nanoplatform by incorporating the hypoxia reliever atovaquone (ATO) with BSL-1 and 17-DMAG in ATO/17-DMAG/BSL-1, which can enhance PTT/PDT via alleviation of thermal resistance and hypoxia [139]. The ATO/17-DMAG/BSL-1 can be efficiently uptake by hepatoma cells from the glyco-targeting by BSL-1, followed by GSH-responsive release of the loaded ATO and 17-DMAG into the cytoplasm (Figure 7). The nanoagent can enhance PTT/PDT synergistically upon 685 nm NIR laser irradiation by using ATO to reduce O_2_ consumption under a hypoxic TME and 17-DMAG to downregulate HSP90 expression, which can enhance the phototherapeutic efficacy of PTT and PDT both in vitro and in vivo.

#### 4.1.5. HSP90 Inhibitor: Purine-Based Inhibitor BIIB021

In addition to natural inhibitors (6-chloro-9-[(4-methoxy-3,5-dimethyl-2-pyridinyl) methyl]-9H-purin-2-amine)) (BIIB021), a synthetic HSP90 inhibitor has also attracted the attention, which is a purine derivative and acts as an N-terminal HSP90 inhibitor. It is the first synthetic inhibitor to enter the clinical trial and can be administered orally. BIIB021 is known to inhibit P-glycoprotein (P-gp) and MRP-1 present in tumor cells [140,141,142]. For nanomaterial-based BIIB021 delivery, Zhang et al. have formulated a mitochondria-targeted nano-micelle by the self-assembly of PEGylated IR-780, which acts as a PTA, with BIIB021 to prepare PEG-IR780-BIIB021 for mild PTT [143]. The nano-micelle can be passively accumulated in MCF-7 cancer cells and selectively enriched in the mitochondria, followed by BIIB021 release upon NIR irradiation. This leads to diminished heat tolerance from HSP90 inhibition as well as reduced mitochondrial membrane potential and downregulated expression of key intrinsic cell anti-apoptotic proteins, which all contribute towards enhanced mild-temperature PTT.

#### 4.1.6. HSP70 Inhibitor: Ver-155008

The Ver-155008 compound can bind to the nucleotide-binding site of HSP70 and arrest the nucleotide-binding domain (NBD) to act as an ATP-competitive inhibitor of HSP70 [144]. It is observed that Ver-155008 can restrict the proliferation of different cancer cells and inhibit tumor growth by obstructing the PI3K/AKT/mTOR and MEK/ERK signaling pathways by directly targeting HSP70 [145]. Tang et al. have proved the advantage of nanomaterial-based Ver-155008 delivery can achieve mild hyperthermia at 45 °C through HSP70 inhibition, which is similar to high-temperature hyperthermia conducted at 55 °C in vitro. To deliver Ver-155008 by nanomaterials, they combined methoxy-polyethylene glycol-SH (MPEG-SH) modified gold nanorod (AuNR) and Ver-155008-loaded methoxy-polyethylene glycol-poly(D, L-lactic acid) micelles (Ver-M) to prepare the nano-drug MPEG-AuNR@VER-M by self-assembly. The nanocomposite could be accumulated in the tumor site from fluorescent imaging and photoacoustic imaging, which can attenuate the heat-resistance of HCT116 cancer cells by acting as an HSP70 N-terminal inhibitor [146]. It was observed that a linear relationship exists between the concentration of Ver-155008 used and the extent of HSP70 inhibition, even at a short laser irradiation time of 1 min, where MPEG-AuNR@VER-M could raise the temperature to 42 °C and enhance the therapeutic outcomes of mild-temperature PTT in vivo.

#### 4.1.7. HSP70 Inhibitor: Phenylethynesulfonamide (PES)

The 2-phenylethynesulfonamide (PES), which is also known as Pifithrin-µ (PFT-µ), is a small molecular inhibitor competing for the C-terminal SBD of HSP70. It can disrupt the binding of HSP70 to its cofactor proteins like HSP40 as well as client proteins like apoptotic peptidase activating factor 1 (a key protein in the apoptosis regulatory network), tumor suppressor protein p53, and autophagy-related protein p62. The mechanism to induce tumor cell death by PES is caspase-independent and occurs through increased protein aggregation, dysfunction of the lysosomal system, proteasomal pathway, and an indirect effect on HSP90 activity [104,147].

You et al. combine NIR-responsive NO-generation gas therapy with HSP70-inhibition-enhanced mild PTT by using a SiO_2_-coated gold rod (Au@SiO_2_). This PTA was sequentially coated with S-nitrosothiols (SNO), (a thermosensitive donor of NO) encapsulated with PES to reduce cancer cell heat resistance, and finally decorated with PEG to enhance biocompatibility and stability [148]. After endocytosis by MCF-7 cancer cells, the PEG−PAu@SiO_2_−SNO nanoparticle can generate heat to induce cell apoptosis/necrosis at low temperatures from PES-mediated HSP70 inhibition and trigger NO release upon NIR light exposure for synergistic mild PTT and gas therapy. Both in vitro and in vivo studies revealed the collaborative anti-cancer efficiency with effective inhibition of the growth of breast cancer cells from arrested HSP70 expression.

**Figure 8:**
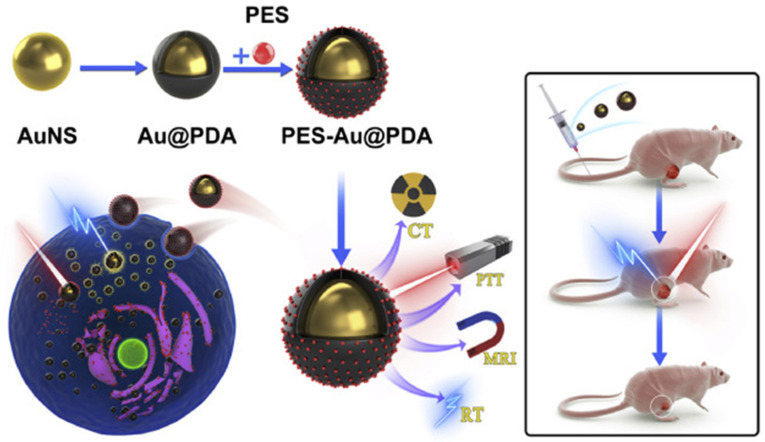
Pifithrin-μ incorporated in gold nanoparticles amplifies pro-apoptotic unfolded protein response cascades to potentiate synergistic glioblastoma therapy. Reprinted with permission from [149]. Copyright 2020, Elsevier Ltd.

A different nanoplatform based on HSP70 inhibition was developed by Zhu et al. by coating a gold nanosphere (AuNS) (a radiosensitizer) with polydopamine (PDA) (a PTA) and PES to enhance the multimodal treatment efficacy of radiotherapy (RT) and PTT [149]. This nanoagent can also be used for monitoring the progression of cancer therapy using computer tomography (CT) and magnetic resonance imaging (MRI) (Figure 8). In this design, PES was employed to inhibit HSPA5, a member of the HSP70 family, by amplifying the pro-apoptotic unfolded protein response (UPR). By incorporating PES, the nanoparticle triggered the UPR cascades and improved the therapeutic efficacy of PTT and RT against the human glioblastoma cancer cell line SW1783 in vitro and in vivo.

#### 4.1.8. HSP70 Inhibitor: Apoptozole

Apoptozole is a small-molecule inhibitor of HSP70 by specifically interrupting the protein-protein interactions and thus inhibiting substrate binding to the SBD of HSP70 [150]. Cui et al. and team members developed a layer-by-layer self-assembled nanodiamond (ND)-based nanoplatform to treat breast cancer via a combination of mild PTT and chemotherapy [151]. Through ND-based autophagy inhibition and accelerated therapeutic efficacy through depletion of transcriptional regulator nuclear protein 1 (NUPR1), the ND-based nanoplatform was constructed by using protamine sulfate (PS)-modified ND to encapsulate ICG (a PTA) and apoptozole. The nanoparticle surface was then coated with HA to prepare PS@ND (ICG + APZ) (NPIA) and loaded with DOX through physical adsorption to prepare NPIAD. On exposure to NIR laser, released apoptozole enhances the efficiency of combination therapy towards triple-negative breast cancer cells through ICG-mediated mild-temperature PTT and ND-mediated chemotherapy via autophagy regulation.

Searching for a small-molecule PTA with high photothermal conversion efficiency (PCE), Ni et al. first developed C6TI as a new type of PTA by adopting a double bond-based molecular motor concept [152]. When the double bond is twisted upon irradiation, the excited molecule can be deactivated through photoinduced nonadiabatic decay to show a PCE as high as 90.0% for low-temperature PTT. To strengthen mild PTT by reversing the thermal resistance of tumor cells, an apoptozole-doped C6TI nanoparticle was prepared through nanoprecipitation and modified with a cell-penetrating peptide derived from the trans-activating transcriptional activator (Tat) to fabricate C6TI/Apo-Tat nanoparticles for targeting the 4T1 tumor cells.

In a recent study, Sun et al. developed a nano-assembly from Pluronic F-127, a non-ionic triblock copolymer, to load apoptozole (APO) and Cy7-2-dicyanomethylene-3-cyano-4,5,5-trimethyl-2,5-dihydrofuran (Cy7-TCF) [153]. The APO/Cy7-TCF@F127 nanoassembly could generate heat under NIR light irradiation and kill cancer cells at a moderate temperature with successful HSP70 inhibition by apoptozole.

#### 4.1.9. HSP70 Inhibitor: LY294002

LY294002 is a PI3k inhibitor and can downregulate the expression of HSP70 [146,154]. Song et al. used Bi2S3–Tween 20 nanodots as a PTA to load LY294002 and used this nanoagent for synergistic HSP70 inhibitors and mild PTTs of tumors [155]. The Bi2S3–Tween 20@LY294002 nano-therapeutic drug delivery system can increase drug utilization rates and reduce side effects on normal tissues. Under low-power NIR irradiation, the Bi2S3–Tween 20@LY294002 can efficiently induce heat generation with the support of LY294002 by downregulating the expression of HSP70. This leads to an obstructed PI3K/Akt/GSK-3/HSP signal transduction pathway and increased tumor cell sensitivity to hyperthermia and activates the BAX/BAK-regulated mitochondrial dysfunction and cell death.

#### 4.1.10. HSP70 Inhibitor: Quercetin

Quercetin is a bioflavonoid widely distributed in fruits and vegetables and exhibits antimicrobial, antioxidant, antiviral, and anti-inflammatory properties [156]. Quercetin is also a potential inhibitor of HSP70 by preventing HSP70 overexpression indirectly by interrupting the phosphorylation of HSF1 [157]. Quercetin phenylisocyanate was reported to trigger apoptosis in tumor cells through inhibition of HSP70 synthesis and expression [158]. Integrating thermosensitive liposomes (Lipos) and quercetin (QE), Arpan et al. encapsulated gold (Au)-coated QE in thermosensitive liposomes to prepare QE-LiposAu [159]. The QE-LiposAu could be used as a PTA with 75% PCE after being exposed to 750 nm NIR laser light. Most importantly, QE-LiposAu showed enhanced photothermal effects to kill Huh-7 hepatocellular carcinoma cells compared to LiposAu nanoparticles without QE. This nanosystem was shown to boost PTT with enhanced cell apoptosis and depolymerization of the microtubule through HSP70 downregulation upon exposure to NIR irradiation.

Hierarchical carbon nanocages emerge as a promising second near-infrared (NIR-II) PTA with a high specific surface area, porous structure, and good photothermal properties. Wang et al. formulated a photothermal-responsive nanosystem by immobilizing quercetin (Q) on a hierarchical nitrogen-doped carbon nanocage (hNCNC) to prepare QhC, followed by functionalization with Zr-based metal-organic hydrogels (MOGs) to form QhC@MOG thermal agent for PTT [160]. On exposure to 1064 nm wavelength NIR light, the MOGs undergo a dry-gel transformation and create pores on their surface to release the loaded quercetin, which can sensitize cancer cell responses to heat through HSP70 inhibition and stimulate the efficiency of PTT. Through dual utilization of hyperthermia and thermal-driven quercetin release, the nanosystem can provide enhanced tumor ablation with mild laser light exposure in vivo.

A summary of the nanomaterial-based approach to enhance cancer photothermal/photodynamic and combination therapy using small-molecule inhibitors of HSP90 and HSP70 is provided in Table 1.

### 4.2. HSP Inhibition via Direct ATP Suppression Mediated by Glucose Deprivation

Although small-molecule HSP inhibitors can serve the purpose of downregulating HSP overexpression, they may have a limited impact on PTT/PDT since they can only transiently bind to specific sites in HSP, where regeneration of HSP activity may occur to reduce the inhibition effect [138,160]. As a result, it was suggested that a shortage in the energy/nutrient supply to the tumor (i.e., hindering the energy source for ATP production) can also affect tumor cell proliferation by modulating HSP overexpression and enhancing PTT and PDT.

Tumor cells have a high demand for nutrients in the form of glucose, which is associated with their rapid progression as compared to normal cells. The process of anaerobic glycolysis converts glucose to lactic acid along with the production of ATP molecules. According to the Warburg effect, cancer cell proliferation occurs via the intake of ATP generated from the glycolysis pathway rather than from the oxidative phosphorylation pathway; therefore, consuming endogenous glucose can cause tumor starvation, which provides a new strategy for cancer therapy termed starvation therapy (ST). Although starvation can happen either by reducing the blood supply or by cutting down the energy supply to tumor cells, the strategy employed for HSP inhibition is glucose deprivation to directly suppress ATP production from the metabolic pathways [161,162]. Glucose oxidase (GOx)-mediated ST is a widely accepted anti-tumor technique that is based on the function of this oxidoreductase to catalyze the oxidation of β-D-glucose to gluconic acid and produce hydrogen peroxide (H_2_O_2_), thus blocking ATP production [163]. However, since GOx is a homodimer protein consisting of two non-covalently bound flavin adenine dinucleotides (FAD), it can induce an immune response. Thus, modification of GOx to decrease its immunogenicity is a significant concern, especially when considering diverse biological applications. Entrapping GOx into a polymer-like matrix is one strategy for reducing its immunological response. Polypyrrole (Ppy) is highly biocompatible for biomedical applications [164]. One study analyzed the stability, conformational changes, and activity of GOx encapsulated in Ppy nanoparticles from fluorescence-based assays [165]. In the process of reactions catalyzed by GOx, fluorescence is observed in the FAD. By utilizing this fluorescence characteristic of FAD, the study examined the stability of GOx. The findings indicate that GOx encapsulated within Ppy nanoparticles demonstrates enhanced stability. The protective Ppy shell effectively prevents the denaturation of GOx, as opposed to free GOx, which undergoes unfolding. This is supported by the fluorescence spectra of FAD during the denaturation of GOx.

By loading glucose oxidase (GOx) in porous hollow Prussian Blue nanoparticles (PHPBNs) and coating them with hyaluronic acid (HA) via redox-cleavable linkage for specific binding with CD44-overexpressing tumor cells, Zhou et al. developed the PHPBNs-S-S-HA-PEG@GOx nanoplatform to combine ST and low-temperature PTT for the treatment of hypoxia tumors [166]. Based on the biocatalytic activity of GOx to deprive endogenous glucose and cut down ATP generation, the efficiency of PTT is enhanced as HSP production is susceptible to ATP depletion. After successful endocytosis of PHPBNs-S-S-HA-PEG@GOx with the enhanced permeability and retention (EPR) effect through PEGylation and efficient active targeting ability mediated by HA, the disulfide bonds present on PHPBNs could be cleaved by GSH intracellularly to release GOx. The released GOx can restore oxygen supply by the decomposition of intratumoral H_2_O_2_ and enhanced glucose depletion to suppress the expression of HSP and reduce cancer cell resistance to low-temperature PTT. By reinforcing tumor thermal ablation through reduced HSP expression by restricting ATP supply, mild PTT with ST was easily combined in this study after NIR laser irradiation.

With their easy synthesis and good biocompatibility, semiconducting polymer nanoparticles can harvest all kinds of electromagnetic irradiation and emerge as functional nanomaterials for PTT/PDT [167]. By exploiting the unique characteristics possessed by cancer cells, such as a low H_2_O_2_ level, a high GSH level, and a mild acidic condition, Hao et al. formulated a Fe_3_O_4_-DOX@PDA-GOx@HA nanosystem for the combination of enhanced chemodynamic/chemo/photothermal/starvation therapy [168]. The nanoagent can be selectively internalized by cancer cells due to HA and release the loaded Fe_3_O_4_, DOX, and GOx. After intracellular uptake, the nanoparticle can trigger multiple reactions, including Fe^3+^ oxidizing the ROS scavenger glutathione (GSH) to glutathione disulfide (GSSG) and GOx catalyzing the production of H_2_O_2_ via glycolysis to generate cytotoxic hydroxyl radical •OH and boost the efficacy of chemodynamic therapy (CDT) from iron-based Fenton-like reactions. Most importantly, the diminished ATP production is shown to affect HSP overexpression, thus improving the mild PTT offered by a biocompatible semiconducting polydopamine layer (PDA) outside the Fe_3_O_4_. Yu et al. constructed a triple-responsive nanoagent based on BZA, a complex synthetic product of near-infrared-II (NIR-II) photothermal reagent Bi–Au, 2-methylimidazole, Zn^2+^, and chloroauric acid [169]. The BZA was functionalized with Gox and tannic acid to form BZAGTA, which can target the tumor cells and provide glycolysis-regulated ST, H_2_O_2_-mediated CDT, and mild PTT. On reaching the tumor site and with the combined effects of ATP, GSH, and acidic TME, the nanoparticle releases Gox and BZA. The GOx can consume glucose produced from the glycolysis reaction for ST and produced H_2_O_2_ from this reaction can be converted to cytotoxic hydroxyl radicals for CDT, while oxygen generated from the reaction catalyzed by BZA can replenish the GOx-mediated glycolysis. Thus, with the combined effect, NIR irradiation at 1064 nm can combine ST, CDT, and enhanced mild-temperature PTT through the glucose consumption-induced thermal sensitization strategy.

Zhu et al. designed a multifunctional nanoreactor to overcome the heat-triggered resistance from PTT through GOx-mediated HSP inhibition, which also provides cancer ST. By enclosing Au nanorod (AuNR) and GOx into a zeolitic imidazolate framework-8 (ZIF-8), the nanoparticle is surface coated by the erythrocyte membrane (eM) for overcoming the cell self-defense mechanism to synthesize ZIF@GOx@AuNRs@eM [170]. On NIR irradiation at 808 nm, the photothermal response from AuNR occurs simultaneously with intratumoral glucose depletion by GOx to inhibit ATP production, which can augment PTT by suppressing the production of HSP. Similarly, Li et al. used a precipitation method to prepare a PTA by self-assembling poly-5,5′-(2,5-bis(2-octyldo-decyl) 3,6-di(thiophen-2-yl)- 2,5-dihydropyrrolo [3,4-c] pyrrole-1,4-dione (PDPP) and 1,2-distearoyl-sn-glycero-3-phosphoethanolamine-N-carboxy(polyethylene glycol)2000 (DSPE-PEG_2000_-COOH) into NIR-light-absorbing conjugated polymer nanoparticles (CPNs) possessing semiconductor properties. The GOx was conjugated to the surface carboxyl of the CPNs through its amino groups to prepare CPNs-G to enhance the mild-temperature photothermal effect via HSP inhibition [171]. By generating local heat and increasing tumor temperature upon 808 nm laser irradiation, the catalytic activity of GOx could be enhanced by impeding the generation of ATP. This NIR light-regulated GOx-mediated nanocatalytic starvation effect can downregulate HSP production and overcome the thermal resistance in PTT by combining ST with mild-temperature PTT.

**Figure 9:**
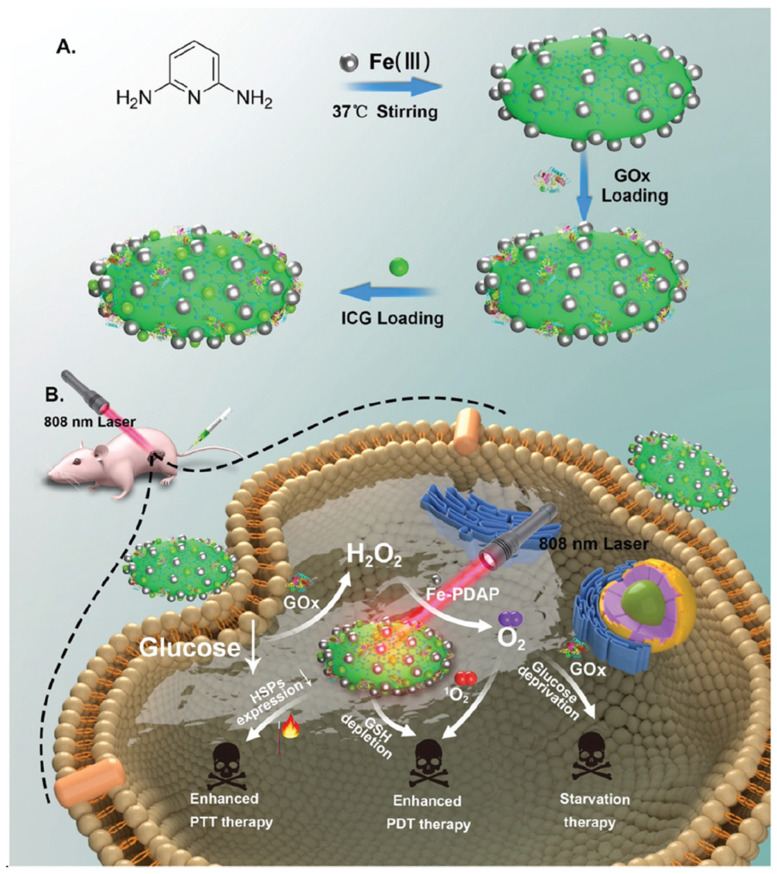
A multimodal imaging-guided nanosystem for the cooperative combination of tumor starvation and enhanced mild-temperature photothermal therapy (PTT) and photodynamic therapy (PDT). Reproduced with permission from [174]. (**A**) Procedure for preparing the nanosystem. (**B**) Schematic illustration showing the multilevel mechanism of the nanosystem. Copyright 2020 Royal Society of Chemistry.

Another study by Wu et al. reports a similar strategy to reverse tumor cell thermoresistance by exploiting the catalytic behavior of GOx in HSP regulation. They used PEGylated CaCO_3_ nanoparticles to encapsulate antimonene quantum dots (AQDs) as a PTA and GOx to engineer a pH-sensitive nanocatalyst G/A@CaCO_3_-PEG system, which can also be used for photoacoustic imaging [172]. With laser light absorption within the NIR window, GOx can restrict the production of ATP along with the deceleration of the expression of HSP to augment hyperthermia-based mild PTT. Image-guided photonic hyperthermia was demonstrated in vivo using tumor-bearing mice. In a different work by Xia et al., the group used two thermosensitive liposomes: GOx-loaded liposome (GOx@Lipo) for ST, and organic NIR dye 1,1′-dioctadecyl-3,3,3′,3′-tetramethylindotricarbo cyanine iodide (DiR)-loaded liposomes (DiR@Lipo) for PTT and PDT [173]. After co-administration of GOx@Lipo and DiR@Lipo to the tail veins of mice bearing 4T1 tumors plus 808 nm laser light irradiation, the combination ST/PTT/PDT shows excellent tumor inhibition via downregulation of heat-induced production of HSP. Interestingly, they also demonstrated that ST could inhibit tumor metastasis through an immunogenic effect. Another multimodal image-guided nanosystem aiming at the combined effects of ST/PTT/PDT was developed by Cao et al. using Fe-doped polydiaminopyridine (Fe-PDAP), an excellent biocompatible organic polymer nanoagent showing catalase-like activity, to load ICG and GOx [174]. In Fe-PDAP/GOx/ICG, the GOx catalyzes the reaction to exhaust glucose for ST, as well as relieving the heat resistance of cancer cells by improving the efficiency of PTT from HSP reduction. Most importantly, Fe-PDAP can replenish the oxygen supply by decomposing intrinsic and GOx-produced H_2_O_2_ to alleviate hypoxia and boost O_2_-dependent PDT (Figure 9). The nanoparticle can substantially improve the outcomes of mild-temperature PTT and PDT by ablating tumors and showing minimal systemic toxicity and multimodal imaging capability.

As stated before, GOx-induced ST is based on the re-programming of metabolic pathways in cancer cells, which is vital for their proliferation, growth, and survival. Aside from GOx, there are alternative ways to prevent the glycolytic process from producing ATP. One such method is the use of lonidamine (1-(2,4-dichlorobenzyl)-H-indazole-3-carboxylic acid, LN), a drug that can particularly block the glycolysis pathway by obstructing the glycolytic enzyme hexokinase II. The delivery of LN by nanomaterials can also modulate ATP production and is expected to downregulate the ATP-dependent production of HSP [175]. Based on this strategy, Shu et al. used hollow mesoporous Prussian Blue (PB) as a PTA to encapsulate LN and DL-menthol (DLM) [176]. After coating with cancer cell membrane (CCM), the PBLM@CCM nanoparticles show a heat-induced phase transition to enable the heat-induced release of LN. On laser irradiation, PBLM@CCM generates heat for mild temperature (≤45 °C) PTT, which also mediates LN release and impedes ATP production. This inhibits HSP production and overcomes the heat endurance of cancer cells to improve PTT.

As the increase in glucose uptake is an important hallmark of cancer cells, another way to interrupt ATP production is to downregulate the glucose transporters. The glucose transporter 1 (Glut1) is such a protein present in the cell membrane lipid bilayer. Expression of Glut1 on the cancer cell surface can be inhibited by resveratrol, which is an antioxidant that exerts an antiproliferative effect on cancer cells and provides a promising target in drug development aimed at treating neoplastic growth [177]. Chen et al. engineered a nanoplatform by incorporating diclofenac (DC) as a Glut1 inhibitor to target CD44-overexpressed cancer cells. Gold nanorod (GNR) was used as a PTA and HA-conjugated DC was used to decorate the GNR surface to prepare GNR/HA-DC nanoparticles [178]. On targeted delivery of the nanoparticle to the tumor site, the hyaluronidase-mediated DC release takes place and depletes the expression of Glut1. A cascade effect takes place during cellular metabolism to inhibit glucose uptake, decrease ATP production, and hamper HSP expression. This leads to diminished protective effects of heat stress experienced by cancer cells and ultimately sensitizes them to PTT.

A summary of the nanomaterial-based approach to enhance cancer photothermal/photodynamic and combination therapy by inhibiting HSP overexpression through glucose deprivation is provided in Table 2.

## 5. Conclusions and Outlook

Among emerging cancer therapies, phototherapy involving PTT and PDT is a prominent and promising approach. However, PTT and PDT can experience certain constraints that restrict their expected efficacy, and a combination of PTT/PDT can escalate the therapeutic effect. Certain irrepressible factors inherent within the TME may hamper the outcomes of PTT/PDT as the self-protective mechanism from cancer cells can be activated under hyperthermia conditions to produce molecular chaperones known as HSP, which renders cancer cells insusceptible to heat stress in PTT. Furthermore, the upregulated production and release of HSP can also be induced by PDT and influence its therapeutic outcomes. As HSP is ATP-dependent, employing small molecules to inhibit ATP binding to HSP or depriving glucose to minimize ATP production from the metabolic pathway can inhibit the activity of HSP and improve the efficacy of PTT/PDT. Alternatively, the use of siRNA technology to downregulate HSP expression can also be combined with PTT/PDT for this purpose. Integrating any of these strategies with nanomaterials by delivering a small molecule for inhibition of the HSP activity or exhaustion of the glucose concentration can uplift the limitations of PTT/PDT. For this purpose, different functionalized nanomaterials have been designed for targeted delivery of a small-molecule HSP inhibitor or a glucose-deprivation nanoagent to reduce the heat resistance of cancer cells and enhance the therapeutic efficacy of mild-temperature PTT. In addition, other components in the TME can be focused on during photothermal treatment. This has led to the development of functional nanomaterials that can easily combine HSP downregulation-based PTT with other cancer therapeutic methods such as CT, CDT, and ST for combination cancer therapy. A desirable feature of these functional nanomaterials will be the possibility for multimodal imaging, which can facilitate image-guided cancer therapy in a clinical setting.

## Figures and Tables

**Table 1:** The nanomaterial-based approach to enhance cancer photothermal/photodynamic and combination therapy using small-molecule inhibitors of HSP90 and HSP70.

Therapy	Nanoagent	HSP Inhibiting Agent	Cancer Cell Line	Reference
PTT	BGP	GA	MCF-7, T47D	[112]
PTT	PGNP	GA	HepG2, H22	[113]
PTT	Mn-ICG@pHis-PEG/GA	GA	4T1	[114]
PTT/CT	HSA/dc-IR825/GA	GA	A549	[115]
PTT/CT/Gas Therapy	PSGHD	GA	WSU-HN6	[116]
PTT/PDT/CT	AuNS@ZrTCPP-GA@LP	GA	4T1, MDA-MB-231	[117]
PTT/PDT/CT	PTX-IR780-GA	GA	4T1	[118]
PTT/PDT	IR780-GA-MnO_2_ (IGM)	GA	4T1	[119]
PTT/CT	BDPII-gel@TSL	Geldanamycin	HeLa	[122]
PTT/PDT/CT	BSA/Cy7–SQ/GM	Geldanamycin	HepG2, MCF-7	[123]
PTT/CT	ICG−17AAG@HMONs−Gem-PEG	17-AAG	MDA-MB-231	[126]
PTT/CT	DOX-17AAG@B-PEG-cRGD	17-AAG	MDA-MB-231	[127]
PTT	iRGD-W_18_O_49_-17-AAG	17-AAG	MKN-45P	[128]
PTT	M-17AAG-AIPH	17-AAG	MDA-MB-231	[129]
PTT/CT	T-GNR_AAG_	17-AAG	HepG2	[130]
PTT/PDT	A/I@aZIF@AAG@HA	17-AAG	LNCaP	[131]
PTT/PDT	17AAG@P(2PMI-AQ)	17-AAG	4T1	[132]
PTT/PDT	17AAG@HMONs-BSA-IrO_2_-PEG	17-AAG	MDA-MB-231	[133]
PTT/CT	17-DMAG/MPNPs@PM	17-DMAG	MCF-7	[137]
PTT/Ferroptosis	17-DMAG-HMPB@sPP@HA	17-DMAG	B16	[138]
PTT/PDT	ATO/17-DMAG/BSL-1	17-DMAG	HepG2	[139]
PTT	PEG-IR780-BIIB021	BIIB021	MCF-7	[143]
PTT	MPEG-AuNR@VER-M	Ver-155008	HCT116	[146]
PTT/Gas Therapy	PEG−PAu@SiO_2_−SNO	PES	MCF-7	[148]
PTT/Radiotherapy	PES-Au@PDA	PES	SW1783	[149]
PTT/CT	NPIAD	Apoptozole	MDA-MB-231	[151]
PTT	C6TI/Apo-Tat	Apoptozole	4T1	[152]
PTT	APO/Cy7-TCF@F127	Apoptozole	HeLa	[153]
PTT	Bi_2_S_3_–Tween 20@LY294002	LY294002	LoVo	[155]
PTT	QE-LiposAu	Quercetin	Huh-7	[159]
PTT	QhC@MOG	Quercetin	MCF-7	[160]

**Table 2:** The nanomaterial-based approach to enhance cancer photothermal/photodynamic therapy and combination therapy by inhibiting HSP overexpression through glucose deprivation.

Therapy	Nanoagent	HSP Inhibiting Agent	Cancer Cell Line	Reference
ST/PTT	PHPBNs-S-S-HA-PEG@GOx	GOx	HepG2	[166]
ST/PTT/CDT/CT	Fe_3_O_4_-DOX@PDA-GOx@HA	GOx	4T1	[168]
ST/PTT/CDT	BZAGTA	GOx	4T1	[169]
ST/PTT	ZIF@GOx@AuNRs@eM	GOx	HCT116	[170]
ST/PTT	CPNs-G	GOx	MCF-7	[171]
ST/PTT	G/A@CaCO_3_-PEG	GOx	SW1990	[172]
ST/PTT/PDT	GOx@Lipo+DiR@Lipo	GOx	4T1	[173]
ST/PTT/PDT	Fe-PDAP/GOx/ICG	GOx	MDA-MB-231	[174]
ST/PTT	PBLM@CCM	Lonidamine	4T1	[176]
ST/PTT	GNR/HA-DC	Diclofenac	HeLa	[178]
